# Autistic adults’ views and experiences of requesting and receiving workplace adjustments in the UK

**DOI:** 10.1371/journal.pone.0272420

**Published:** 2022-08-05

**Authors:** Jade Davies, Brett Heasman, Adam Livesey, Amy Walker, Elizabeth Pellicano, Anna Remington

**Affiliations:** 1 UCL Centre for Research in Autism and Education (CRAE), University College London, London, United Kingdom; 2 School of Education, Language and Psychology, York St John University, York, United Kingdom; 3 Neurodiversity Works, London, United Kingdom; 4 Macquarie School of Education, Macquarie University, Sydney, Australia; University of Warsaw, POLAND

## Abstract

This article examines 181 autistic adults’ views toward, and experiences of, requesting and receiving workplace adjustments in the UK. Using an online survey, we collected both qualitative and quantitative data relating to individuals’ experiences. While the majority of participants perceived workplace adjustments to be important, many were not receiving them. Analysis of open-ended text responses highlighted specific challenges that autistic people face in requesting and receiving adjustments. Specifically, participants felt the onus fell on them to (*1*) identify their need for adjustments; (*2*) establish the specific adjustments that would be beneficial and (*3*) request adjustments from their employer. Yet, they reported struggling with this process. Participants also highlighted a range of social and organisational barriers to the successful implementation of workplace adjustments. Unsurprisingly, the lack of successfully implemented adjustments had far-reaching impacts on participants’ wellbeing as well as the choices they made about their future employment. These findings highlight the need for employers to take a more active role in the identification and implementation of workplace adjustments, as well as a need for more understanding and inclusive working environments that truly allow autistic employees to thrive in the workplace.

## Introduction

Workplace adjustments enable employees with specific needs to work safely and productively, and facilitate equal participation and better employment opportunities for disabled people [1–3]. Adjustments may include modifications to the physical environment, processes of communication, working hours and/or job roles [4]. For example, adjusting the lighting above one’s workstation, avoiding the use of idioms and figurative speech, or modifying work hours to avoid a rush-hour commute. In the United Kingdom (UK), employers are required to offer ‘reasonable adjustments’ under the Equality Act 2010 [5]. Under this legislation, any disabled employee is entitled to ‘reasonable adjustments’ if they are considered to be at a substantial disadvantage, relative to employees that are not disabled, without the adjustment. According to the Act, what is considered ‘reasonable’ should be determined by the employer, and may depend on individual organisational factors such as financial resources. In order to access adjustments, employees are expected to disclose their disability and provide evidence that they are at a substantial, and not ‘minor’ or ‘trivial’, disadvantage compared to their non-disabled counterparts. While a formal diagnosis of a disability is not legally required, employers are able to request medical evidence of a disability and can reject requests for adjustments on the basis that they do not believe an employee meets the criteria to be considered disabled [6].

Autistic people face higher unemployment rates than other disability groups in the UK [7] and are therefore one group of people for whom adjustments can be crucial. Indeed, evidence suggests that the implementation of workplace adjustments can support autistic people in accessing work and allow employers to make use of the unique skillset autistic people can offer [8]. Yet, research in the United States (US) suggests that autistic people can struggle to access workplace adjustments [9, 10]. There is also a limited body of research on autistic individuals’ experiences of workplace adjustments, which means that little is known about the most effective workplace adjustments for autistic people [11]. The current research seeks to address this gap by gathering the views and experiences of autistic adults on workplace adjustments across a range of organisations and industries.

There are several reasons why autistic people may not successfully receive workplace adjustments. First, employers often lack sufficient knowledge and understanding of autism to identify the adjustments that autistic people require at work [12]. This lack of knowledge may be further exacerbated by the fact that autism is often ‘hidden’ from others’ view, making individuals’ needs unclear to employers. As such, individual employees are expected to “make the case” for their adjustment requests [13], thus placing the burden on them to (1) identify the need for an adjustment and (2) request the necessary adjustments from their employer [14]. Yet, making such a case can be a challenging process. For example, research with people with visual impairments shows that employees are often unaware of their specific adjustment needs, their adjustment options, or even their right to ‘reasonable adjustments’ [15]. Similarly, research in the US suggests that autistic employees may also struggle to understand which adjustments may be of use to them [16]. As such, autistic employees may find it challenging to identify both their need for an adjustment and the appropriate adjustment to request.

Second, once adjustment needs have been identified, obtaining the desired modifications involves speaking up and negotiating with employers. The former involves employee voice: verbal or written communication in which an employee makes a choice about whether to speak up about their concerns [17]. Yet, speaking up about one’s concerns involves a complex communication process [18]. For example, research with autistic employees suggests that many autistic people perceive diagnostic disclosure as a necessity in order to gain access to adjustments [19]. Yet, evidence from the broader disability literature suggests that diagnostic disclosure is contingent on the adoption of a disability identity [20]. This is problematic as the journey to developing a disability identity can be complex and lengthy [21], and may mean that those who do not have a strong autistic identity are discouraged from requesting the adjustments they require. Indeed, autistic employees often experience difficulties in social communication at work [22, 23] and, as such, even those with a strong autistic identity may find communicating their needs with their employers particularly challenging.

Furthermore, research on the factors associated with speaking up about safety concerns in organisational cultures suggests that the process is also contingent on a series of individual factors such as self-confidence and extraversion [24]. However, autistic people can be anecdotally shy, introverted and lacking in confidence [25, 26]. Indeed, autistic people have historically faced oppression [27] and, perhaps as a result, often experience internalised ableism–when disabled people internalise society’s prejudices and act accordingly [28]. Consequently, autistic people may not possess the skills or confidence to approach an employer about their needs. This lack of confidence to approach employers about one’s needs may be further exacerbated by additional group factors. For example, research suggests that the act of requesting adjustments can be perceived as confrontational by employers [29] and diagnostic disclosure can lead to autistic people being perceived as less helpful [30]. As such, autistic people may be reluctant to address their adjustment needs. Indeed, a recent systematic review examining autistic adults’ experiences of requesting and receiving adjustments suggests that up to 50% of autistic employees do not request workplace adjustments [11]. This reluctance is not unfounded: research suggests that the process of negotiating workplace adjustments with employers can have dire consequences on employee wellbeing [31].

Third, when individuals are able to speak up about their needs, adjustments are not always successfully implemented. For example, a study examining the implementation of ‘reasonable adjustments’ across different disabilities in the UK found that 30% of requested adjustments were not implemented [32]. Similar figures are also seen in the US [33]. The literature on disability and workplace inclusion has suggested a range of potential social and organisational factors that contribute to the gap between adjustment requests and adjustment implementation. Suggested social factors include the characteristics of the individual requesting the adjustments and the impact of adjustments on others within the organisation [34, 35], while suggested organisational factors include the size of the organisation, workplace adjustment policies in place and the cost of the adjustment to the organisation [36–38]. It is not known, however, whether (and how) these factors affect the implementation of workplace adjustments for autistic individuals specifically.

Fourth, even if implemented, workplace adjustments are not always effective. For example, research in the UK indicates that even when adjustments are agreed upon, they are often delayed [31] or inconsistently implemented [39]. As a result, employers often perceive the impact of workplace adjustments more favourably than autistic employees themselves. Overall, the process of requesting and receiving adjustments is both personal and complex. Autistic people are expected to advocate for their own adjustment needs yet many may not feel that they have the tools to be able to do so. Given the high unemployment rates that autistic people face [7] and the fact they often struggle to sustain long-term employment [40, 41], it is important for research to establish how both employees and employers can best identify and implement sustainable ‘reasonable adjustments’ that encourage job satisfaction and consequent employee retention.

The current study, therefore, sought to establish the workplace adjustments autistic people require, and the barriers they face in requesting and receiving such adjustments. To address this aim, we gathered the first-hand perspectives of employees on the autistic spectrum using a short online survey. Listening directly to autistic adults themselves is crucial if research is to identify the factors that contribute to the successful implementation of adjustments. Indeed, research shows that when ‘reasonable adjustments’ are successfully and consistently implemented, outcomes are positive [42].

## Methods

This study was part of a broader programme of research exploring autistic adults’ employment experiences using the Diverse Minds Survey. The Diverse Minds Survey is an online survey that gathers information about neurodiversity and employment undertaken in the UK. The survey was developed in collaboration with autistic reviewers, as part of the Discover Autism Research and Employment (DARE) project, and was advertised through three main channels, including (1) Autistica’s Discover Network for autistic people interested in taking part in research; (2) the research team’s social media channels, and (3) invitations to organisations that had approached the project and expressed an interest in understanding more about neurodiversity and employment.

### Participants

Participants in this study were autistic adults (over 18 years of age) based in the UK, with experience of employment. To be included in the present study, participants needed to (1) identify as autistic; (2) be currently, or previously, employed, and (3) have completed both the demographic survey and at least 50% of the module-specific survey on workplace adjustments within the Diverse Minds Survey.

When the data were extracted in March 2020, a total of 220 participants had navigated to the adjustments section of the Diverse Minds Survey, and were included in the final analyses. While the views and experiences of self-identified autistic employees are valid and important, few participants self-identified as autistic in our sample (*n* = 12). Given that the legal right to ‘reasonable adjustments’ is often perceived to be associated with having a formal diagnosis, we expect the adjustment experiences of autistic individuals with a formal diagnosis, and those without, to be different. The small sample of self-identified autistic employees meant, however, that we were unable to make meaningful comparisons between the two groups and therefore chose to exclude self-identified participants. We will endeavour to explore the unique experiences of this group in future research, using a larger sample. An additional 27 cases were removed for not having reported any experiences of employment (*n* = 12, 44.4%), not having completed the full demographics questionnaire (*n* = 10, 37%), or not having completed at least half of the workplace adjustments module (*n* = 5, 18.5%). The final sample comprised 181 participants. The sample was not representative of the wider autistic population. For example, the majority of the participants included reported being of a white ethnic background (*n* = 153, 84.5%) and/or identified as female (*n* = 108, 59.7%). All participants were aged between 18 and 75 years. The majority of participants were in full-time (*n* = 69, 38.1%) or part-time (*n =* 40, 22.1%) employment, or were self-employed (*n* = 19, 10.5%). Table 1 reports full demographic information.

**Table 1:** Participants demographic information (*n* = 181).

Background variables	*N*	%
***Gender identity***		
Female (including transwoman)	108	59.7
Male (including transman)	61	33.7
Non-Binary	9	5.0
Other	3	1.7
***Age***		
18–25	19	10.5
26–35	46	25.4
36–45	41	22.7
46–55	53	29.3
56–65	20	11.0
66–75	2	1.1
***Ethnicity*** ^1^		
White	153	84.5
Mixed/Multiple ethnic groups	13	7.2
British/English/UK^1^	12	6.6
Black	1	0.6
South African^1^	1	0.6
Unspecified	1	0.6
***Highest level of education***		
Master’s Degree (e.g., MA, MSc, MEd)	53	29.3
Bachelor’s Degree (e.g., Bsc, BA, BEd)	51	28.2
Vocational qualification (e.g., BTEC, GNVQ, HND)	18	9.9
A/AS Level (qualification at 16–18 years old)	16	8.8
Doctorate	13	7.2
Other postgraduate study (e.g., PGCe, PGDip)	11	6.1
GCSE’s (qualification at 14–16 years old)	6	3.3
Foundation Degree	4	2.2
No formal education	3	1.7
Other (e.g. fellowship to professional body)	5	2.8
***Employment status***		
Employed full-time	69	38.1
Employed part-time	40	22.1
Self-employed	19	10.5
Unemployed (not looking for work)	19	10.5
Unemployed (looking for work)	15	8.3
Student	9	5.0
Retired	6	3.3
Volunteer	4	2.2
***Satisfaction with employment***		
Satisfied	82	45.3
Unsatisfied	49	27.1
Uncertain	40	22.1
Other	7	3.9
N/A	3	1.7
***Size of most recent employer (total number of employees)***		
0–5 employees	11	6.1
6–20 employees	16	8.8
21–50 employees	9	5.0
51–100 employees	13	7.2
101–500 employees	25	13.8
501–1,000 employees	16	8.8
1,001–10,000 employees	31	17.1
>10,000 employees	31	17.1
Unspecified	29	16.0
***Number of past employers***		
None	2	1.1
1–2 employers	21	11.6
2–4 employers	37	20.4
4–6 employers	38	21.0
More than 6 employers	81	44.8
Prefer not to say	2	1.1
***Most recent income***		
< £10,000	40	22.1
£10,000–£19,999	42	23.2
£20,000–£29,999	36	19.9
£30,000–£39,999	22	12.2
£40.000–£49,999	10	5.5
£50,000–£59,999	4	2.2
£60,000–£79,999	7	3.9
£80,000–£99,999	4	2.2
£100,000–£149,999	4	2.2
Prefer not to say	12	6.6
***Highest level worked at***		
Intern, apprentice or volunteer	12	6.6
Entry level/graduate employment	65	35.9
Mid-level employment	68	37.6
Senior-level employment	27	14.9
Prefer not to say	9	5.0
***Most common employment sectors***		
Education	27	14.9
Healthcare	20	11.0
Public sector	16	8.8
IT	13	7.2
Administration	12	6.6
Retail	9	5.0
Engineering	8	4.4
Charity	7	3.9

^1^Note: this question had a free text response option. As such, some participants did not report their ethnicity, and instead reported their nationality.

### Materials

All participants that took part in the Diverse Minds Survey completed a general module providing demographic and employment information (e.g. employment status, number of previous employers, estimated income). Participants in this study also completed a module regarding their experiences of requesting and receiving workplace adjustments. The workplace adjustments module comprised closed-ended questions regarding (1) whether adjustments had been requested and/or implemented; (2) the type of adjustments requested; (3) participants’ experience of discussing their adjustment needs, and (4) the perceived importance of workplace adjustments. Participants were also invited to answer open-ended questions about their personal experiences of adjustments, probing for what contributed to their success (or lack thereof) and what their perceptions were of the organisational decision-making process surrounding implementing workplace adjustments. See S1 File for a copy of the survey.

### Procedure

Ethical approval was obtained through the Research Ethics Committee at UCL Institute of Education, Faculty of Education and Society (REC1149). All participants provided written consent to take part and the workplace adjustments questionnaire took approximately ten minutes to complete.

Quantitative data are presented descriptively (*n*, %). Responses to the open-ended question “what examples have you experienced of successful or unsuccessful adjustments?” were analysed using qualitative content analysis [43]. JD independently coded the adjustments named in responses in alignment with those presented on the multiple choice question “what type of adjustments have you asked for?”. JD then categorised these adjustments as either successful or unsuccessful, and generated a frequency table of responses. The remaining qualitative data were analysed using iterative categorisation [44] with a view to generate domain summary themes about autistic employees’ experiences of workplace adjustments. BH conducted an initial analysis of the data, taking an inductive, open-coding approach (i.e., forming categories without a pre-existing coding framework) to segment the data. From the responses, it was clear that experiences extended beyond simply receiving adjustments, instead involving a more complex process around (*1*) identifying adjustment needs, (*2*) the implementation of adjustments and (*3*) the subsequent outcomes when adjustments are/are not successfully implemented. Accordingly, BH grouped the open codes together inductively to form three main categories, with relevant sub-categories, ensuring that every unit of data (i.e., every sentence about adjustment experiences) could be accounted for. JD also independently coded the responses according to the above categories and sub-categories, refining where necessary. All authors agreed on the final set of categories and sub-categories. Inter-rater reliability was high both at the category (k = 0.93) and subcategory (k = 0.81) levels.

## Results

### Quantitative results

#### Experiences of requesting and discussing adjustments with employers

Despite the majority of participants perceiving workplace adjustments as extremely or very important (*n* = 152, 83.9%), just over half (*n* = 106, 58.6%) reported requesting them. Only a small minority of participants felt adjustments were not necessary for them (*n* = 10, 5.5%). Encouragingly, of the 106 participants who *had* requested adjustments, 65 (61.3%) reported them being implemented. A sizeable number of other participants, however, had their adjustment requests either refused (*n* = 27, 25.5%) or poorly implemented (*n* = 14, 13.2%). Almost one-third of the participants (*n* = 57, 31.5%) reported that they had not requested adjustments but felt that they would have been beneficial for them. A further five participants (2.8%) reported that they had not requested adjustments themselves but that their employer had suggested and successfully implemented workplace adjustments on their behalf. See Table 2 for a comprehensive breakdown of participants’ experiences of requesting workplace adjustments.

**Table 2:** Participants’ experiences of requesting adjustments (*n* = 181).

		*n*	%
**Requested adjustments (*n* = 106, 59%)**	I have asked for adjustments and these have been made for me	65	35.9
	I have asked for adjustments but have been refused	27	14.9
	I have asked for adjustments but they were not properly implemented	14	7.7
**Did not request adjustments (*n* = 73, 40%)**	I have not asked for adjustments but they would have been beneficial	57	31.5
	I don’t feel that adjustments are necessary for me	10	5.5
	My employer suggested adjustments for me and they were successful	5	2.8
	I did not know I required adjustments	1	0.6
**Other (*n* = 2, 1%)**	Unsure if I have requested adjustments	1	0.6
	I am self-employed and make my own adjustments	1	0.6

Participants’ experiences of discussing adjustments with employers were varied. Many participants felt able to discuss their needs either with a trusted colleague (*n* = 69 of 151, 45.7%), with specific individuals they felt needed to know (e.g., Occupational Health) (*n* = 4 of 151, 2.6%) or more freely within the whole organisation (*n* = 26, 17.2%). Yet, almost one-third of the participants (*n* = 47 of 151, 31.1%) reported feeling unable to discuss their adjustment needs. Five participants (of 151, 3.3%) reported trying to discuss their adjustment needs with an employer but receiving an unsatisfactory response, such as not being taken seriously or having the adjustments implemented incorrectly.

#### Types of adjustments requested

The 106 participants that reported requesting adjustments also shared details regarding the types of workplace adjustments they requested (see Table 3). Of these participants, 78 (73.6%) reported requesting *changes to the physical environment and equipment*; 76 (71.7%) requested *changes to their job role and supports*, and 70 (66.0%) requested *changes to social and cultural practices*.

**Table 3:** Workplace adjustments participants reported requesting (*n* = 106).

Category	Examples	*n*	%^1^
**Changes to physical environment and equipment**	Access to new equipment or permission to use own equipment (e.g. noise-cancelling headphones), software to improve accessibility, designated quiet spaces, allocated desk or office, allocated car parking space	78	73.6%
**Changes to job role and supports**	Evolving job role based on strengths; flexible work hours to avoid commuting in rush hour; remote working where possible; additional supports (e.g. information resources, mentors)	76	71.7%
**Changes to social and cultural practice**	Changes to communication (e.g. explicit communication, asking one question at a time, advanced notice of changes); changes to social obligations; increased understanding and training on neurodiversity; flexibility regarding clothing choice where possible	70	66.0%

^1^Note: percentages exceed 100% as categories were not mutually exclusive.

Of the 106 participants that indicated they had requested adjustments, 81 reported on their perceived success (see Table 4). The majority of those (*n* = 54, 66.7%) reported on the success of *adjustments to the physical environment*. Over half (*n* = 34, 63.0%) reported finding such adjustments successful, with access to new equipment and access to an allocated desk or office space being perceived as particularly successful. Forty-eight participants (59.3%) reported on the success of *adjustments to their job role and accompanying supports*. Of those, 41 participants (85.4%) reported finding such adjustments successful. Finally, 33 participants (40.7%) reported on the success of *adjustments to social and cultural practice*. The perceived success of these adjustments was mixed: 18 participants (54.5%) found them successful and 19 (57.6%) did not.

**Table 4:** Successful and unsuccessful workplace adjustments (*n* = 81).

Changes to physical environment (*n* = 54, 66.7%)		
	Successful (*n* = 34, 63.0%)^1^	Unsuccessful (*n* = 26, 48.1%)^1^
Access to new equipment or use of personal equipment (e.g., noise-cancelling headphones)	19 (55.9%)	6 (23.1%)
Allocated desk or office space	13 (38.2%)	3 (11.5%)
Designated quiet space	7 (20.6%)	13 (50.0%)
Changes to sensory environment (e.g., lighting, noise)	5 (14.7%)	9 (34.6%)
Changes to clothing	2 (5.9%)	0 (0.0%)
Changes to job role and supports (*n* = 48, 59.3%)		
	Successful (*n* = 41, 85.4%)^1^	Unsuccessful (*n* = 10, 20.8%)^1^
Changes to working hours	19 (46.3%)	5 (50.0%)
Remote working	13 (31.7%)	0 (0.0%)
Changes to job role or responsibilities	9 (22.0%)	5 (50.0%)
Having a mentor or advocate	4 (9.8%)	1 (10.0%)
Having additional breaks or additional time for tasks	3 (7.3%)	0 (0.0%)
Changes to social and cultural practices (*n* = 33, 40.7%)		
	Successful (*n* = 18, 54.5%)^1^	Unsuccessful (*n* = 19, 57.6%)^1^
Changes to communication	14 (77.8%)	12 (63.2%)
Changes to organisational culture	4 (22.2%)	5 (26.3%)
Changes to understanding	1 (5.6%)	5 (26.3%)

^1^Note: percentages exceed 100% as categories were not mutually exclusive

#### Diagnostic disclosure

Although diagnostic disclosure was not the focus of this research, we also examined participants’ experience of requesting and receiving workplace adjustments upon such disclosure. Of the 181 participants that took part in the current study, 160 (88.4%) responded to the Diverse Minds Survey module on diagnostic disclosure, with almost all (*n* = 149 of 160, 93.1%) indicating that they had disclosed their diagnosis to at least one colleague. Of the 11 participants who chose not to disclose their diagnosis, only four (36.4%) had requested adjustments, with the remaining seven (63.6%) indicating that they had not requested adjustments but they might have been beneficial. Many of these participants (*n* = 8, 72.7%) reported feeling unable to discuss adjustments with employers.

#### Qualitative results

Data from the open-ended survey questions were organised into a series of subcategories that formed the basis of three main categories: (*1)* challenges in identifying adjustments; (*2*) perceived factors impacting the implementation of adjustments and (*3*) outcomes of poor implementation or refusal of adjustments. See Table 5 for a breakdown of each category.

**Table 5:** Factors related to the experience of workplace adjustments (*n* = 121).

Categories	Sub-categories	*N* (%)
**Challenges in identifying adjustments *(n* = 13, 10.7%)**	Employers are not aware of their employees’ support needs or respective adjustments	10 (8.3%)
	Employees had difficulties in identifying their own adjustment needs or respective adjustments^1^	4 (3.3%)
**Perceived factors impacting the implementation of adjustments *(n* = 114, 94.2%)**	Practical considerations (e.g., finances, resources, time, feasibility)	90 (74.4%)
	The impact of adjustments on others	39 (32.2%)
	The impact of individual, employee factors	25 (20.7%)
	The impact of manager traits and understanding	16 (13.2%)
	Stigma associated with requesting adjustments	14 (11.6%)
	Understanding of legal obligations	12 (9.9%)
	Social status and responsibility of the organisation	5 (4.1%)
	Unable, or unwilling, to disclose one’s diagnosis	5 (4.1%)
**Outcomes of poor implementation or refusal of adjustments *(n* = 28, 23.1%)**	The need for continuous advocacy	12 (9.9%)
	Implications for mental health	10 (8.3%)
	Changes to employment status (e.g., terminating employment, turning to self-employment)	7 (5.8%)
	Unfair dismissal	4 (3.3%)
	Need for external guidance and support	3 (2.5%)

^1^Note: while participants had a formal diagnosis at the time of completing the survey, difficulties in identifying adjustment needs were often reported as being experienced prior to the diagnosis.

#### Challenges in identifying adjustments

Many participants reported challenges in identifying possible adjustments, which was perceived as a barrier to successful employment. Specifically, participants felt employers were unaware of employees’ potential support needs, as well as the corresponding adjustments to address those needs: “*No-one seems to actually understand sensory issues*” (Participant 004; henceforth, P004). Participants felt this was a particular issue for autistic employees as: “*the nature of a ’disability’ being hidden allows for a person’s challenges to be overlooked*, *leading to a lack of consideration [from employers]*” (P056).

As a result of the perceived gaps in employer knowledge and understanding of workplace adjustments, autistic employees felt that “*the onus was on me to identify what adjustments could be made*” (P001). Yet, some participants reported difficulties in identifying their need for adjustments (“*I haven’t made many requests*, *because I didn’t know I had a clinical reason for feeling uncomfortable*”; P128) or the specific adjustments that may be particularly beneficial for them: “*I find it difficult to pinpoint what adjustments or support I might need*, *and feel further guidance regarding this and what is available to help would be very helpful*” (P151).

#### Perceived factors impacting the implementation of adjustments

In cases where individuals were able to identify their support needs and appropriate corresponding adjustments, participants reported several barriers that they felt prevented adjustment requests from being successfully implemented. To begin, some participants did not feel comfortable disclosing their diagnosis, making it difficult to broach the subject of workplace adjustments: “*I haven’t disclosed so it’s difficult to talk about asking for adjustments*. *I wish I could*” (P112). Yet, even those that did feel able to communicate their need for adjustments faced challenges: “*[my employers] were dismissive of my concerns and laughed when I was upset*, *saying it ‘couldn’t really be all that bad’*. *I felt they negated my experience and distress and invalidated and dehumanised me*” (P154). Similarly, some participants reported facing stigma when asking for adjustments; for example, being “*told I was trying to be a problem*” (P117) and being “*made to feel I am being really difficult*” (P036).

Requesting and receiving reasonable adjustments were also perceived to be linked with employee identity and how individuals were perceived and valued by their employers and colleagues. For example, individual employee factors such as the perceived status of the employee (“*could the person be replaced if adjustments cannot be met*?”; P066) and the impact of the adjustments on others within the organisation (“*will any changes have a detrimental impact on the other employees*?”) (P119) were commonly reported as impacting manager decision-making regarding adjustments.

Other, more external, factors were also perceived to influence the likelihood of adjustments being successfully implemented. For example, practical considerations were highly cited (*n* = 90 of 121, 74.4%) and included resource factors such as cost, convenience and feasibility of adjustments: “*[employers] own convenience and money*” (P126). Participants also highlighted the impact of the individual receiving the request on the likelihood of adjustments being implemented: “*It depends how knowledgeable*, *compassionate*, *bothered [management] are*” (P002). Similarly, participants considered the social status of the organisation (“*[employers consider] would doing the adjustments improve our image*?”; P149) and their understanding of their legal obligations (“*[employers consider] the law … and potential discrimination claims*”; P111) as important factors potentially impacting the implementation of adjustments.

#### Outcomes of poor implementation or refusal of adjustments

Participants who successfully received workplace adjustments perceived them as beneficial: “*[Before receiving adjustments]*, *I would become irritated*, *overwhelmed and frustrated*. *The immediate moment ear defenders were offered*, *my productivity slowly improved*” (P107). However, difficulties in successfully receiving workplace adjustments were not uncommon, with participants reporting they had to prove adjustments would be beneficial for their requests to be considered. For some participants, this involved seeking external guidance: “*any adjustments were made several years after diagnosis and via evidence provided by an NHS Psychologist and Psychiatrist*” (P080). Yet, regardless of whether they had been provided with this additional guidance, some participants reported that they were still refused their workplace adjustments or had their adjustments poorly implemented. Indeed, some participants noted that even if adjustments were implemented, they often had to continue to advocate to keep their adjustments (“*[I was] given an allocated desk space but I had to fight for it back after it was taken away without warning*”; P007), which was perceived to have major implications on employees mental health: “*it wears away at me*, *corrodes my mind*” (P143). In some cases, the lack of adjustments led to a termination of employment (“*I have not had any unsuccessful adjustments provided*, *rather the refusal to accommodate my challenges altogether*. *This has either resulted in sick leave or quitting the job*”; P056) and/or moving towards other, more sustainable forms of employment: “*After years of losing jobs*, *I decided that the only way I can work is to be self-employed*, *I have never had a full time job where enough adjustments could be made to make the job sustainable for me*” (P005). Other individuals reported being “*managed out*” (P001) of their employment or, in some cases, unfairly dismissed: “*I asked if I could reduce my hours*, *work less people-facing and more admin based but was refused and ultimately fired for not being able to fulfil the role*” (P103).

## Discussion

Using a bespoke online questionnaire, we gathered the views and experiences of autistic adults requesting and receiving workplace adjustments. While the majority of participants in this study perceived workplace adjustments to be important, many reported not receiving them. Qualitative analyses highlighted the specific challenges that our participants faced when requesting and receiving adjustments. First, participants reported challenges in identifying both their need for adjustments and the specific adjustments that might best support them. Yet, even when they were able to identify their needs, many people reported facing additional barriers to the successful implementation of adjustments. These barriers included social barriers such as fear of disclosure, stigmatisation, the perceived status of the individual requesting adjustments and the impact of adjustments on others within the organisation. Other, more organisational, barriers to successful implementation included the availability of organisational resources, manager views and traits, and the social status of the organisation. Unsurprisingly, the lack of successfully implemented adjustments was perceived to have far-reaching impacts on the participants’ wellbeing as well as the choices they made about their future employment. Here, we discuss how these findings relate to existing research and provide recommendations for future research and adjustment practices.

Participants in the current study felt that they would benefit from knowledgeable, understanding employers that could guide them through the process of requesting and receiving workplace adjustments. Unfortunately, this need for supportive employers was not often met, and employers were often perceived as unknowledgeable regarding workplace adjustments. As such, the onus fell on autistic individuals to (*1*) identify their need for adjustments, (*2*) specify appropriate adjustments and (*3*) make adjustment requests. This finding is in line with existing research from the US, suggesting that employees often have to “make the case” for their adjustments [13–15]–something with which our participants clearly struggled, for several reasons. First, they were not always aware that they required adjustments. Second, those that were able to identify the need for adjustments were not always able to specify precisely which adjustments might be beneficial. This difficulty specifying adjustments coincides with research with individuals with visual impairments which found that employees are not always well-informed of their adjustment needs, the kinds of adjustments available and the process of requesting adjustments [15]. While these findings are not necessarily unique to autistic individuals, it is important to note that such barriers are likely to be exacerbated for autistic adults, who themselves often report experiencing additional communicative challenges [23]. Indeed, participants in this study reported specific challenges in making adjustment requests and many faced additional social barriers (e.g., stigmatisation) when they did request workplace adjustments.

In order to improve the experiences of autistic individuals requesting workplace adjustments, organisations should seek to promote a more inclusive workplace culture that embraces workplace adjustments. Based on our participants’ responses, we suggest a two-pronged approach, involving (1) informing employers and (2) empowering employees. First, organisations should provide managers with training about the adjustments available within their organisation, and their benefits, as well as the protocol for implementing workplace adjustments. The provision of specific autism training to all non-autistic employees was also recommended by our participants, especially including autistic employees in the design and delivery of this training, and providing colleagues with relatable examples and information. Where possible, organisations should utilise materials that provide employees with relatable and memorable examples of autistic individuals’ experiences and their potential need for adjustments (see, for example, [45]). Second, organisations should seek to empower employees to request workplace adjustments by providing them with clear guidance regarding the adjustments that are available, and how they can request them. This might include, for example, having a dedicated space on the company intranet containing examples of the adjustments available, and information regarding how employees can request adjustments. Similarly, organisations may seek to develop Disability Employee Resource Groups that can empower individuals to advocate for adjustments and ensure employees get equal access to workplace adjustments.

Many participants also noted challenges with the perceptions of others within their organisation. For example, autistic people often felt they were labelled as “troublesome” or “unreasonable” when they requested adjustments, with their employers not believing they had any genuine need for workplace adjustments. Yet, evidence suggests that individuals with physical disabilities do not face the same kind of stigma when requesting adjustments [46]. This may be a reflection of the fact that autism is a ‘hidden disability’ and as such is less likely to be picked up on, and understood, by employers [47]. As a result, many participants felt unable to request adjustments without disclosing a formal autism diagnosis. This finding is consistent with recent findings by Romualdez et al. [19], who found that disclosure was perceived as a necessity by autistic employees, as opposed to a choice (see also, [11]). However, the requirement to provide information about one’s diagnosis in order to gain access to necessary workplace adjustments is problematic for several reasons. First, it excludes self-identifying autistic individuals from requesting, and thus receiving, the adjustments they need. Second, it creates a double-edged sword whereby autistic individuals are forced to make a choice between keeping their diagnosis private and gaining access to workplace adjustments. Disclosure may not be a goal for all autistic people and a truly inclusive workplace should not necessarily require individuals to disclose in order to receive the adjustments that they require. Instead, organisations should strive to be more active in their role of identifying, suggesting and providing workplace adjustments. One potentially less stigmatizing recommendation may be to provide *all* employees with information about the adjustments the organisation is willing and able to offer, as well as the tools and resources they need in order to make adjustment requests, regardless of their diagnosis or disability. Indeed, evidence from a recent study in China found that taking an identity-blind approach to workplace adjustments, that is providing all employees regardless of disability status equal access to workplace adjustments, had beneficial impacts for both disabled and non-disabled employees [48].

Finally, our findings outline a disparity between the number of individuals desiring, requesting and successfully receiving workplace adjustments. While more than 80% of our participants valued workplace adjustments, less than 60% reported requesting them, and more than one-third of those who *had* requested adjustments did not have them successfully implemented. This finding replicates those from other disability groups where approximately 30% of adjustment requests are not implemented [32, 33]. It is critical to understand why this implementation gap exists, in order to ensure that employees get access to the adjustments they need. This is particularly salient for autistic people, who face much higher unemployment rates than other disability groups [7]. Participants in this study provided insights into the factors they perceived to impact the implementation of adjustments, which largely mapped onto those that have been reported within the broader disability and workplace inclusion literature. These factors included organisational ones, such as the cost of adjustments to the organisation, and social ones such as the perceived status of the individual and the impact of adjustments on others (see for example, [34]). However, it is not yet clear whether any barriers identified, such as stigmatisation and manager traits, are unique to autistic individuals, or, more broadly, individuals with ‘hidden’ disabilities. Future research may seek to conduct a comprehensive review of the barriers and facilitators impacting the implementation of workplace adjustments, and identify any factors that are specific for autistic employees.

### Limitations

This research is not without its limitations. First, it is important to note that this research was carried out prior to the outbreak of the coronavirus. As a direct result of the COVID-19 pandemic, employees across the globe have experienced changes in their working practices (e.g., more remote working, changes to working hours). As such, it is possible that experiences of requesting and receiving workplace adjustments have changed. For example, organisations may now be more receptive to implementing certain adjustments (e.g., flexible working) and some adjustments may have been enforced by Government, and thus not need requesting (e.g., remote working). Future research should seek to examine autistic employees’ experiences of workplace adjustments in light of the COVID-19 pandemic.

Second, our sample may not be representative of the UK autistic population in two key ways. In regard to demographic representation, most of our participants were well-educated, female and reported being of white ethnic background and, as such, this research only represents a sub-group of the autistic population. The lack of cultural diversity is particularly noteworthy given that autistic people of colour may be multiply disadvantaged in the workplace, as minority ethnic groups often face additional workplace inequalities [49]. Future research should aim to address this imbalance by purposively recruiting autistic adults from more varied ethnic backgrounds; for example, by advertising in minority community groups. It is also noteworthy that participants were able to complete a comprehensive online survey which involved reflecting on their workplace experiences. As such, our sample may not represent the views and experiences of autistic people with an intellectual disability, or those who do not use traditional forms of communication. We also did not seek verification of our participants’ autism diagnosis.

Regarding employment experience, almost three-quarters of our participants were in full-time, part-time or self-employment. While this is not consistent with estimates which suggest that as few as 22% to 32% of autistic adults are in paid employment [7, 50], this is likely due to the nature of the survey being based on employment experiences, thus discouraging those who are not employed from participating. Most of our sample also reported working within a medium-to-large organisation (i.e., organisations with 50+ employees). It is possible, however, that larger organisations have access to greater resources to devote to corporate social responsibility practices and policies and support the needs of their employees. Indeed, evidence suggests that larger organisations *are* more likely to implement workplace adjustments than smaller organisations [36]. It is therefore possible that our findings underestimate the experiences of autistic people who work in small organisations.

Finally, we were unable to recruit a large enough sample of self-identified autistic adults to include in the current research. Given the dearth of adult diagnostic services and lengthy waiting lists for those that do exist [51, 52], it is important to include the reflections of individuals that have not had access to a formal autism diagnosis in research. However, it is likely that these two groups, formally diagnosed and self-diagnosed autistic adults, have different experiences of requesting and receiving workplace adjustments as those that self-identify may not be afforded the same legal rights to adjustments as those with a formal diagnosis, or may not be able to provide evidence to their employers regarding the impact of their disability.

Given the limitations highlighted above, we make a number of suggestions for future research. First, it may be of interest to compare the adjustment experiences of non-autistic and autistic employees to establish which findings, if any, are unique to autistic people. Second, researchers may seek to examine the views and experiences of employers of autistic people and/or autistic people’s colleagues, to triangulate the findings and thus gain a fuller understanding of the factors that impact the successful implementation of workplace adjustments for autistic people. Third, we suggest that future research should purposively seek the voices of self-identified autistic people to examine their specific adjustment experiences, and compare them with the experiences of autistic adults with a formal diagnosis (who do and do not disclose their diagnosis). Fourth, it will be important to examine the role of the size of the employment organisation in autistic people’s experiences of requesting and receiving workplace adjustments.

## Conclusion

Notwithstanding the limitations noted above, this study highlights the specific workplace adjustments that autistic people may be likely to request, the kinds of adjustments that they perceive as successful and unsuccessful, and the perceived barriers to their implementation. Importantly, we note that autistic individuals are often faced with the burden of identifying the need for adjustments, identifying the appropriate adjustments and finally requesting the adjustment from their employer. Employers must take a more proactive role in identifying, suggesting and implementing adjustments for their autistic employees, working in collaboration, as opposed to placing the burden solely on the employee. As part of such initiatives, organisations should move away from disclosure as a necessity and provide employees with the information, resources and tools they need to make informed decisions about adjustments, based on their individual needs as opposed to their diagnosis. Future research should review the barriers and facilitators to the successful implementation of workplace adjustments, and establish whether there are any factors unique to autistic employees.

## Supporting information

S1 File Experiences of adjustments. (DOCX)
