# Supplementary material for: Autistic adults’ views and experiences of requesting and receiving workplace adjustments in the UK
Source: PLoS One. 2022 Aug 5;17(8):e0272420. doi: 10.1371/journal.pone.0272420 (PMC9355205; doi:10.1371/journal.pone.0272420)
Supplement: S1 File — (DOCX) [file pone.0272420.s001.docx]

**S1. Adjustments Survey**

**Experiences of adjustments**

Adjustments in the workplace are integral to tailoring employment experiences to individual needs and aptitudes.

Adjustments can take many forms, such as changes to the physical and sensory environment, and changes to line management (e.g. flexible working hours).

In this section, if you feel comfortable to do so, please share your experiences of adjustments that you have encountered.

**Which of the following best describes your experience of your longest employer?**

☐ I have asked for adjustments they were implemented
☐ I have asked for adjustments but have been refused
☐ I have asked for adjustments but they were NOT properly implemented
☐ I have not asked for adjustments but they would have been beneficial
☐ I DON'T feel that adjustments are necessary for me
☐ Adjustments were suggested by my employers and were implemented
☐ Adjustments were suggested by my employers and were NOT implemented
☐ Other (please specify)

**What type of adjustments have you asked for? (select all that apply)**

☐ Changes to equipment (e.g. noise-cancelling headphones)
☐ Changes to working hours
☐ Changes to the physical environment
☐ Changes to job role
☐ Changes to clothing or appearance
☐ Changes to social obligations (e.g., attending dinners_
☐ Changes to logistics (e.g., how to travel)
☐ Changes to supports (e.g., information resources, mentors)

**When starting your most recent role, how long did it take to get adjustments implemented?**

☐ They were in place immediately
☐ 1–3 months
☐ 4–6 months
☐ 7–12 months
☐ More than a year
☐ Adjustments were never implemented

**What examples have you experienced of successful or unsuccessful adjustments?**

___________________________________________________________________________

___________________________________________________________________________

___________________________________________________________________________

**Are there adjustments that you asked to be implemented and were they successful? If not, why were adjustments refused or unsuccessful?**

___________________________________________________________________________

___________________________________________________________________________

___________________________________________________________________________

**What factors do you think organisations consider when deciding whether adjustments are put in place?**

___________________________________________________________________________

___________________________________________________________________________

___________________________________________________________________________

**How important is it to be able to make adjustments in the workplace?**

☐ Extremely important
☐ Very important
☐ Moderately important
☐ Slightly important
☐ Not at all important
